# In Vitro Lethal Effects of Benzyl Benzoate Concentrations of 25%, 10%, and 5% on *Demodex folliculorum* Mites: An Experimental Study With a Control Group

**DOI:** 10.1111/jocd.70175

**Published:** 2025-04-07

**Authors:** İlkay Can, Murat Durdu

**Affiliations:** ^1^ Department of Dermatology, Faculty of Medicine Balıkesir University Balıkesir Turkey; ^2^ Department of Dermatology, Faculty of Medicine Başkent University Adana Turkey

**Keywords:** benzyl benzoate, *demodex folliculorum*, rosacea

## Abstract

**Background:**

Demodex mites have been implicated in the pathogenesis of many dermatologic diseases, especially rosacea. Although many case reports have been published about the treatment of skin diseases caused by *Demodex folliculorum* mite, no relevant treatment algorithm has been developed so far. In this context, we investigated the lethal effects of different doses of benzyl benzoate on this mite.

**Objective:**

To compare the lethal effects of 3 different doses of benzyl benzoate on *Demodex folliculorum* mites with those of a control group consisting of immersion oil.

**Materials and Methods:**

Waste samples collected from rosacea patients were included in the study. Four different groups of 20 mites were formed. Each group was exposed to 5%, 10%, and 25% concentrations of benzyl benzoate, respectively. Immersion oil was used as a negative control group. The movements of the mites were monitored with a digital microscope. The time to death of the mites was recorded.

**Results:**

The mean time to death of mites was 26 ± 2,9, 120 ± 7.6, and 168 ± 15 min after application of benzyl benzoate at concentrations of 25%, 10%, and 5%, respectively. The mean time to death of mites in immersion oil applied as a negative control group was 192 ± 6.4 min. Among the study groups, only the 25% benzyl benzoate group had a statistically significantly shorter mean time to death compared to the control group (*p* = 0.03).

**Conclusion:**

While low concentrations of benzyl benzoate were ineffective in the treatment of demodicosis, benzyl benzoate at high concentrations, e.g., 25%, was found to be effective in its treatment.

## 1 Introduction

**FIGURE 1:**
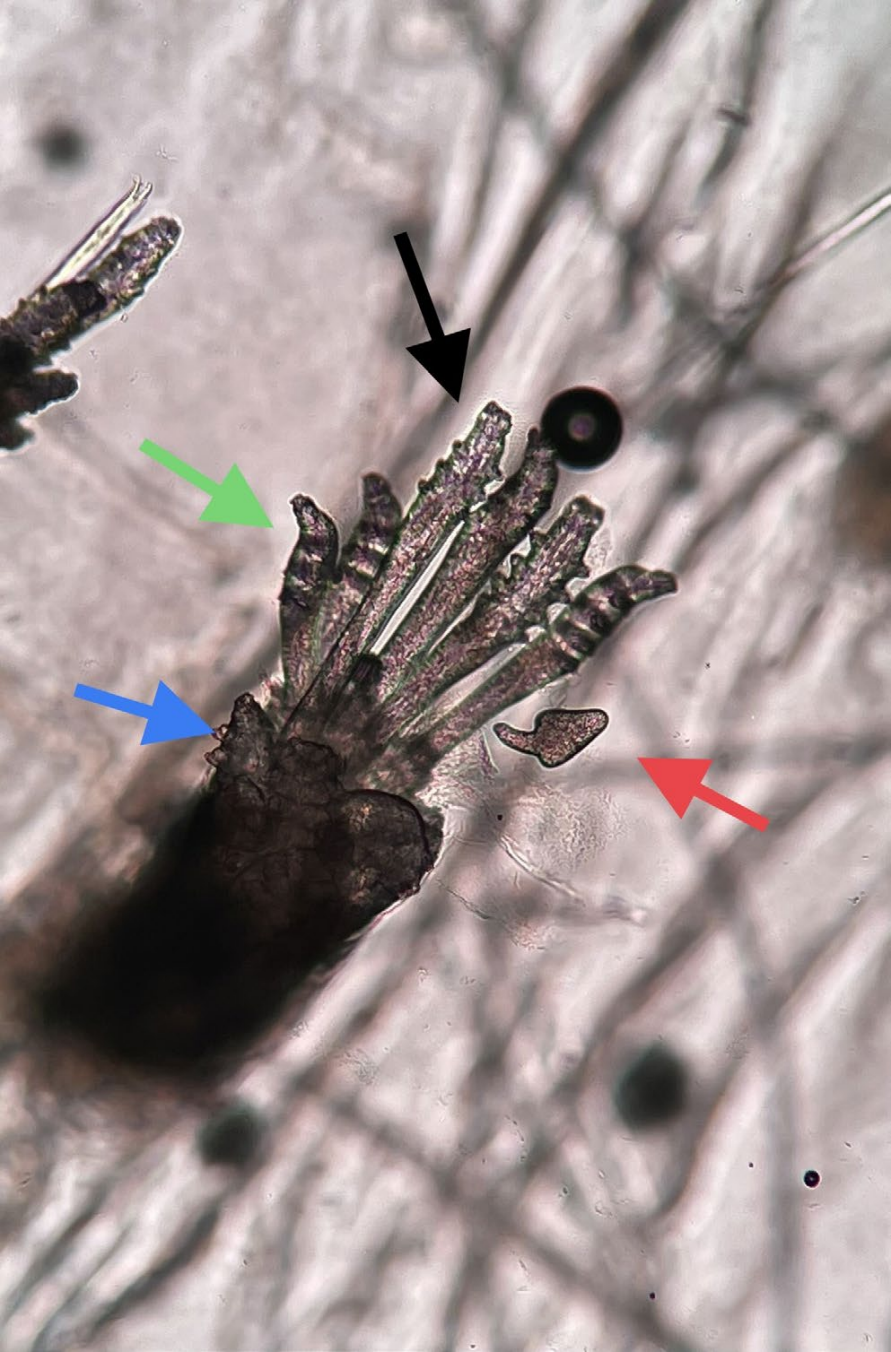
Red arrow: Demodex egg, black arrow: Nymph, green arrow: *Demodex folliculorum*, blue arrow: *Demodex brevis*.

Demodex mites are obligate human parasites and tend to settle in areas where pilosebaceous units are dense. Although there are more than 40 species, only two different species, namely *Demodex brevis* and *Demodex folliculorum*, have been detected in humans. Demodex mites have 4 pairs of legs and a body with a tail. While *Demodex folliculorum* has a longer body appearance compared to *Demodex brevis*, *Demodex brevis* has a cigar‐like shape and a thicker and shorter appearance [1]. The life cycle of the mite is observed in progressive phases of development as egg, nymph, protonymph, and adult (Figure 1). Its average life span is 22 days. Both mite species settle in the hair follicles and feed on sebum residues. *Demodex brevis* settles deeper in the hair follicles than *Demodex folliculorum*. The mites are photophobic and migrate to the skin surface at night.

When we look at the diseases caused by Demodex mite, dermatologic and ophthalmologic diseases come to the forefront. In ophthalmology, increased Demodex density has been associated with many eye diseases, especially blepharitis, and there are many studies on this subject [2]. Although studies in the field of dermatology have been increasing in recent years, there is no clear consensus on clinical types and treatments of demodicosis. It is thought that increased Demodex load rather than the presence of Demodex mites plays a role in the pathogenesis. Demodicosis consists of a series of dermatologic diseases [3]. Chen et al. proposed classifying demodicosis disease as primary and secondary forms associated with immune suppression. In this classification, primary demodicosis includes pityriasis folliculorum (spinulate demodicosis), papulopustular/nodulocystic or conglobate demodicosis, ocular demodicosis causing chronic blepharitis, chalasia or less frequently keratoconjunctivitis, and auricular demodicosis causing external otitis or myringitis. They generally associated secondary demodicosis with systemic or local immunosuppression [4]. Forton et al. proposed classifying demodicosis as inflammatory and non‐inflammatory demodicosis [5].

Although there is no clear consensus, it would be more accurate to consider demodicosis under two headings as primary demodicosis and secondary demodicosis. Primary demodicosis can be considered as the appearance of xerosis, erythema, papular or pustular lesions due to increased Demodex density without any other dermatosis in the background, while secondary demodicosis can be considered as the exacerbation of a pre‐existing disease such as rosacea due to the Demodex load on an already present primary dermatosis such as rosacea. In fact, apart from this conceptual confusion, whether the existing dermatosis regresses or not with antiparasitic treatment carries greater importance. If the existing dermatosis regresses with antiparasitic treatment, it is possible to talk about demodicosis. Since there are case‐based publications on its treatment, a common treatment algorithm has not yet been established. In our study, we aimed to compare the effect of benzyl benzoate on Demodex mites and the acaricidal effects of its different doses.

## 2 Material Method

In our in vitro study, we aimed to observe the reactions of mites exposed to the active substances. Waste samples taken from rosacea patients were included in the study. Benzyl benzoate was applied to the study groups and immersion oil to the control group. In this context, 4 different study groups were formed. Groups were treated with 5%, 10%, and 25% benzyl benzoate concentrations and immersion oil, respectively. The reaction of the mites to the agents was observed, and time‐course of mite death was recorded.

Completely alive and mobile mites were included in the study. Mites that moved both their bodies and limbs were considered fully alive mites. In preparations containing more than one mite, the reaction of the most mobile mite to the agent was observed.

A digital microscope was used for tracking the mites. For better observation of mite movements, the image from the digital microscope was transferred to a larger LCD screen (Video S1). The movements of the mites were observed simultaneously on this screen. The active ingredients were gently injected between the slide and coverslip with an insulin injector. The time from the start of drug exposure to complete paralysis of mites was defined and recorded as the time of death. Mites that remained completely immobilized for 1 min were considered dead. All mites in the study groups were evaluated by the same dermatologist.

### 2.1 Statistical Analysis

The data were analyzed using SPSS (Statistical Package for Social Sciences for Windows v.26.0, SPSS Inc., Chicago, IL). Descriptive statistics were presented as mean (±) standard deviation. The normality of data distribution was assessed using the Shapiro–Wilk test and histogram graphs. Comparisons between groups were performed using the Mann–Whitney *U* test, and a *p*‐value of < 0.05 was considered statistically significant.

## 3 Results

Since the samples taken mostly contained *Demodex folliculorum* mites, the in vitro killing effect of the agents was evaluated on *Demodex folliculorum* mites. In our study, three study groups consisting of 20 mites each and a negative control group were used. The mean time to mite death was 26 ± 2,9 min, 120 ± 7.6 min, and 168 ± 15 min after exposures to 25%, 10%, and 5% benzyl benzoate concentrations, respectively. The mean time to mite death when exposed to immersion oil used as a negative control group was 192 ± 6.4 min.

Among the study groups, only the 25% benzyl benzoate group had a statistically significant shorter mean time to mite death compared to the control group (*p* = 0.03). Although the mean time to death was 26 min, a rapid slowing of movements was observed in the 25% benzyl benzoate group immediately after exposure to the agent.

## 4 Discussion

Demodex mites are ectoparasites that settle in the pilosebaceous unit and feed on sebum residues. Increasing numbers of studies have been performed on these mites in recent years. In ophthalmologic studies, despite the lack of any consensus on this issue, Demodex mites have been blamed in the etiology of blepharitis [2]. Although they are associated with many dermatologic diseases, Demodex mites are primarily implicated in the etiology of rosacea. The most commonly recognized skin diseases caused by Demodex spp. are primary and secondary demodicosis. Primary demodicosis includes diseases that occur due to an increase in the number of demodex mites without any underlying dermatosis. These dermatologic conditions include pityriasis folliculorum (spinulate demodicosis), papulopustular/nodulocystic, or conglobate demodicosis. Secondary demodicosis is an exacerbation seen with an increase in the number of demodex mites in a patient with an underlying dermatosis such as rosacea [4]. What draws attention in the etiopathogenesis of both types of demodicosis is the uncontrolled proliferation and increase in the number of Demodex mites. The most commonly used standard method for the detection of Demodex mites is to examine superficial skin biopsy samples. To speak of increased Demodex density, ≥ 5 Demodex mites per cm^2^ must be observed. Although there is no clear consensus on the role of demodex mites in disease pathogenesis, the most valuable approach that will lead us to a conclusion is whether or not the existing dermatosis regresses with antiparasitic treatment. If the existing dermatosis regresses with antiparasitic treatment, it is possible to talk about demodicosis.

Topical permethrin, metronidazole, crotamiton, benzyl benzoate, ivermectin, tea tree oil, and oral metronidazole are being used in the treatment of demodicosis. There is no algorithm for the treatment of *Demodex* spp. yet. There are case reports of treatment on a case‐by‐case basis. In a study, the in vitro effect of tea tree oil on Demodex parasites was evaluated, and 6 different concentrations of tea tree oil ranging between 2.5% and 100% were investigated. As a result of the study, it was found that all concentrations, including its 2.5% preparation, killed *demodex* spp. within the first 1 h of the treatment [6]. Another systematic review recommends topical tea tree oil as a first‐line treatment for demodicosis [7].

There are very limited publications on benzyl benzoate treatment in cases of demodicosis. There are different approaches, especially regarding the dosage of this agent. Forton et al. used low and high doses of benzyl benzoate in patients with rosacea and demodicosis and reported that high dose benzyl benzoate used at concentrations ranging between 20%–24% gave faster and more effective results than its low dose of 12% [8]. In our study, we observed that benzyl benzoate administered at concentrations of 5% and 10% did not give statistically significant beneficial results when compared with the control group.

In our in vitro study, we directly exposed the mites to various active substances. In order to improve the quality of the study, we used fully alive mites that can move all their limbs and body instead of the previously used concepts of mite vitality that is “moving one limb, moving two limbs”. Thanks to the digital microscope we used and the LCD screen where we transferred the image to, we were able to observe the movements of mites more clearly and objectively.

Our study has shown that the use of low dose benzyl benzoate is ineffective in the treatment of demodicosis. We would like to emphasize that benzyl benzoate at a concentration of 25% can be used as an effective treatment for demodicosis.

## Consent

The authors have nothing to report.

## Conflicts of Interest

The authors declare no conflicts of interest.

## Supporting information


Video S1.

## Data Availability

The data that support the findings of this study are available from the corresponding author upon reasonable request.
